# Photoconductive Gain Behavior of Ni/β-Ga_2_O_3_ Schottky Barrier Diode-Based UV Detectors

**DOI:** 10.3390/mi17010100

**Published:** 2026-01-12

**Authors:** Viktor V. Kopyev, Nikita N. Yakovlev, Alexander V. Tsymbalov, Dmitry A. Almaev, Pavel V. Kosmachev

**Affiliations:** 1 Research and Development Centre for Advanced Technologies in Microelectronics, National Research Tomsk State University, 634050 Tomsk, Russia; nik_mr_x@mail.ru (N.N.Y.);; 2 Department of Radio Equipment Design and Production, Tomsk State University of Control Systems and Radioelectronics, 634050 Tomsk, Russia

**Keywords:** gallium oxide, ultraviolet photodiode, self-powered mode, internal gain

## Abstract

A vertical Ni/β-Ga_2_O_3_ Schottky barrier diode was fabricated on an unintentionally doped bulk (−201)-oriented β-Ga_2_O_3_ single crystal and investigated with a focus on the underlying photoresponse mechanisms. The device exhibits well-defined rectifying behavior, characterized by a Schottky barrier height of 1.63 eV, an ideality factor of 1.39, and a high rectification ratio of ~9.7 × 10^6^ arb. un. at an applied bias of ±2 V. The structures demonstrate pronounced sensitivity to deep-ultraviolet radiation (λ ≤ 280 nm), with maximum responsivity observed at 255 nm, consistent with the wide bandgap of β-Ga_2_O_3_. Under 254 nm illumination at a power density of 620 μW/cm^2^, the device operates in a self-powered mode, generating an open-circuit voltage of 50 mV and a short-circuit current of 47 pA, confirming efficient separation of photogenerated carriers by the built-in electric field of the Schottky junction. The responsivity and detectivity of the structures increase from 0.18 to 3.87 A/W and from 9.8 × 10^8^ to 4.3 × 10^11^ Hz^0.5^cmW^−1^, respectively, as the reverse bias rises from 0 to −45 V. The detectors exhibit high-speed performance, with rise and decay times not exceeding 29 ms and 59 ms, respectively, at an applied voltage of 10 V. The studied structures demonstrate internal gain, with the external quantum efficiency reaching 1.8 × 10^3^%.

## 1. Introduction

Monoclinic β-Ga_2_O_3_ (gallium oxide) has recently attracted significant attention as a promising ultra-wide bandgap semiconductor because of its large bandgap *E*_g_ = 4.5–4.9 eV, high breakdown electric field *E*_br_ = 8 MV/cm, and outstanding Baliga’s figure of merit ≈ 3444, which surpass those of SiC (340) and GaN (870) [1,2,3,4,5]. β-Ga_2_O_3_ substrates can be grown by melt-based bulk growth techniques [6,7], resulting in crystals with low dislocation densities (~10^3^ cm^−2^), high structural quality, and commercial wafer diameters reaching up to ~4–6 inches [1,2,8,9,10]. These technological advantages, combined with its intrinsic electronic properties, position β-Ga_2_O_3_ as a leading material for next-generation power electronic devices, such as Schottky barrier diodes (SBDs), which are key components in energy conversion systems for electric vehicles, air-conditioning, and power distribution [11,12,13,14].

In addition to power electronics, β-Ga_2_O_3_ has emerged as a highly promising material for solar-blind ultraviolet (UV) photodetectors operating in the 200–280 nm wavelength range without the need for optical filters [15,16,17,18,19,20]. The large bandgap ensures intrinsic solar blindness, while its capability for forming high-quality metal–semiconductor interfaces enable the realization of SBD-based photodetectors with low dark current, high responsivity, low power consumption, and self-powered operation [21,22].

Beyond conventional photodetection, β-Ga_2_O_3_ devices also hold potential for functional optoelectronic applications, such as photonic switches and light-controlled electronic components [23]. The ability of β-Ga_2_O_3_ Schottky diodes to combine rectifying behavior typical of power devices with strong photosensitivity under UV illumination makes them suitable candidates for use as photo-controlled switches (photokeys) [24]. Such devices can operate in both forward and reverse bias modes, enabling a tunable balance between sensitivity and signal-to-noise ratio, which is particularly attractive for integrated optoelectronic circuits and intelligent power systems [25].

In the classical description of Schottky barrier diodes, photoelectric gain mechanisms are not explicitly considered. However, numerous recent studies have reported exceptionally high photoelectric characteristics, where the external quantum efficiency exceeds 100%, indicating the presence of internal gain effects [26,27]. The dominant gain mechanism proposed in these works is typically associated with a reduction in the effective Schottky barrier height, caused by the accumulation of photogenerated holes in trap states near the metal/β-Ga_2_O_3_ interface [28]. This charge trapping leads to barrier modulation and enhanced electron injection, resulting in amplified total current. Most studies on Ga_2_O_3_-based photodetectors have focused on device characterization within a limited bias voltage range, often without considering the electric-field dependence of the response and recovery times [29,30]. As a result, the correlation between bias-dependent photoresponse dynamics and internal gain mechanisms remains insufficiently understood. A systematic analysis of these dependencies is essential for developing a more comprehensive understanding of the physical processes governing ultraviolet photoresponse formation in Ga_2_O_3_-based structures.

In this work, the electrical and photoelectric characteristics of Ni/(−201)β-Ga_2_O_3_ Schottky barrier diodes are systematically investigated. It is demonstrated that the fabricated structures exhibit a pronounced photoelectric gain, with the external quantum efficiency exceeding 10^3^%. The phenomenon of photo-gain in SBDs is analyzed over a wide bias voltage range, and the dominant internal gain mechanism responsible for the enhanced photoresponse is identified. The electric-field dependence of the photoconductivity rise and decay times is investigated.

## 2. Materials and Methods

Unintentionally doped β-Ga_2_O_3_ commercial wafers (Tamura Corp., Tokyo, Japan) with a (−201) surface orientation, a thickness of 650 µm, and a donor concentration of ~10^17^ cm^−3^ were used as substrates for the fabrication of vertical Schottky barrier diodes (SBDs). Prior to the formation of the ohmic contact, the substrate surface was etched in hot hydrochloric acid (HCl 30%) and subsequently rinsed in deionized water. Immediately before metal deposition, the surface was cleaned using oxygen plasma.

**Figure 1:**
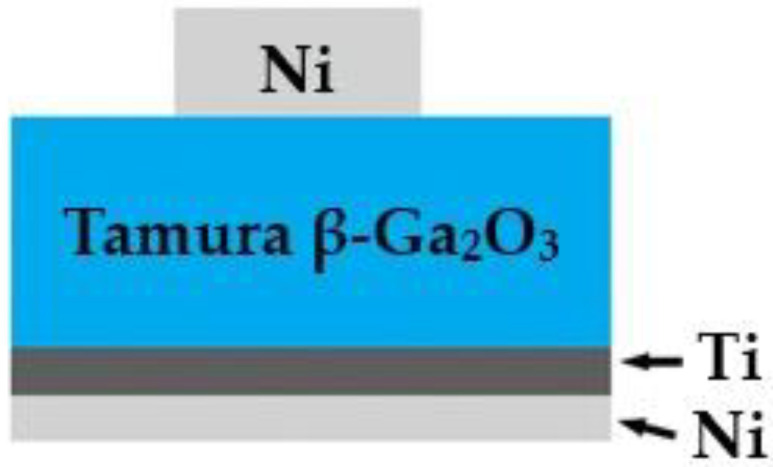
A schematic view of SBD-based on Ni/β-Ga_2_O_3_.

The ohmic contact was formed by electron-beam evaporation of Ti (80 nm) followed by a protective Ni layer (30 nm), and a subsequent annealing process was performed in nitrogen at 300 °C for 30 min. Schottky barrier contacts were patterned using photolithography, and an 80 nm Ni layer was deposited to form the rectifying electrodes. The use of Ni enables a reduction in on-resistance compared to metals such as Pt, Pd, and Ir, which provide higher barrier heights [31,32]. Additionally, Ni is a more cost-effective metal and can be readily deposited via electron-beam evaporation or magnetron sputtering [31,32,33]. The diameter of the Schottky barrier contact is 0.5 mm. In the final step, the substrate was divided into individual 2 mm × 2 mm chips using disk sawing. A schematic of the device structure is shown in Figure 1.

Optical transmission spectra were obtained using a deuterium lamp D-2000, Micropack (Orlando, FL, USA) in the wavelength range of 250–450 nm. The transmitted radiation was collected by an Ocean Optics Flame spectrometer (200–850 nm operating range) with a spectral resolution of 1 nm. Time-resolved photocurrent measurements were performed using a LeCroy 104XS digital oscilloscope (Chestnut Ridge, NY, USA) with 1 GHz bandwidth and a UV LED with a maximum intensity at λ = 255 nm.

Current–voltage (*I*–*V*/*J*–*V*) and capacitance–voltage (*C*–*V*) characteristics were measured using a Keithley 2636A source (Cleveland, OH, USA) meter and an Agilent E4980A RLC meter (Santa Rosa, CA, USA), respectively. *C*–*V* measurements were performed at 1 kHz with an AC signal of 100 mA. The electrical and photoelectric characteristics of the structures were measured under room temperature and ambient-humidity conditions.

A krypton-fluorine lamp VL-6.C (Marne-la-Vallée, France) was used as the source of irradiation at wavelength λ = 254 nm and the light power density *P* = 620 μW/cm^2^. Measurements of the electroconductive characteristics of the SBD were carried out at dark conditions in a sealed Nextron MPS-CHH chamber (Busan, Korea) equipped with microprobes.

Dependencies of the photo-to-dark current ratio (PDCR), responsivity *R*, detectivity *D*, and external quantum efficiency (EQE) of samples on λ and *U* were computed by means of following formulas, respectively [34]:

(1) $$PDCR=\frac{I_{L}}{I_{D}},$$

(2) $$R=\frac{I_{ph}}{S\times P},$$

(3) $$D=R\sqrt{\frac{S}{2eI_{D}}},$$

(4) $$EQE=R\frac{hc}{e\lambda }\times 100\%,$$

where *I*_L_—total current under UV irradiation, *I*_ph_ is the photocurrent, *I*_ph_ = *I*_L_ − *I*_D_; *h* is the Planck constant, *c* is the speed of light in vacuum; *e* is the electron charge, and *S*—diode surface area, *P*—optical power density of the irradiation, and λ—wavelength of the irradiation.

The rise *t*_r_ and the fall *t*_d_ times were determined as the time interval between the 0.9 and 0.1 levels of the maximum *I*_L_.

## 3. Results

**Figure 2:**
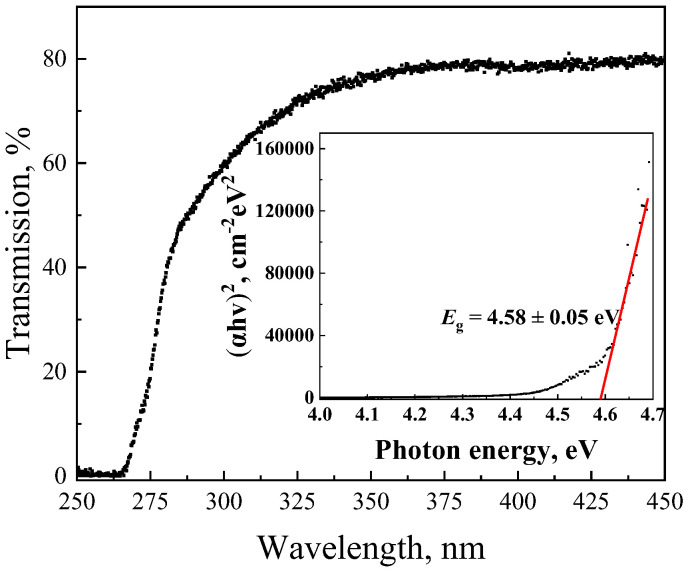
Transmission spectrum of the β-Ga_2_O_3_ bulk (insert: α^2^ versus photon energy).

The optical transmission spectrum of the Ga_2_O_3_ film in the 250–450 nm range shows a strong wavelength dependence typical of a wide-bandgap semiconductor. The transmittance remains very low below approximately 280 nm, indicating strong optical absorption in the deep-UV region where photon energies exceed the bandgap of Ga_2_O_3_. As the wavelength increases beyond 280 nm, the transmission gradually rises, reaching about 80% near 350 nm. The sharp absorption edge observed near 270–280 nm corresponds to the fundamental bandgap absorption of β-Ga_2_O_3_, confirming its suitability for solar-blind ultraviolet photodetection. Based on the measured transmission spectrum, the spectral dependence of the absorption coefficient α was obtained using the following expression [35]:

(5) $$\alpha \left(\lambda \right)=-\frac{1}{d}ln(\frac{\sqrt{(1-R{)}^{4}-4T^{2}R^{2}}-(1-R{)}^{2}}{2TR^{2}}),$$

where *d* is the thickness of the gallium oxide substrate (650 μm), *T* is the transmittance, and *R* is the reflectance. A plot of the squared absorption coefficient (α^2^) versus photon energy (*hν*) was then plotted to determine the optical bandgap (*E*_g_) of the β-Ga_2_O_3_ substrate (Figure 2, inset). The *E*_g_ value for β-Ga_2_O_3_ is 4.58 ± 0.05 eV.

**Figure 3:**
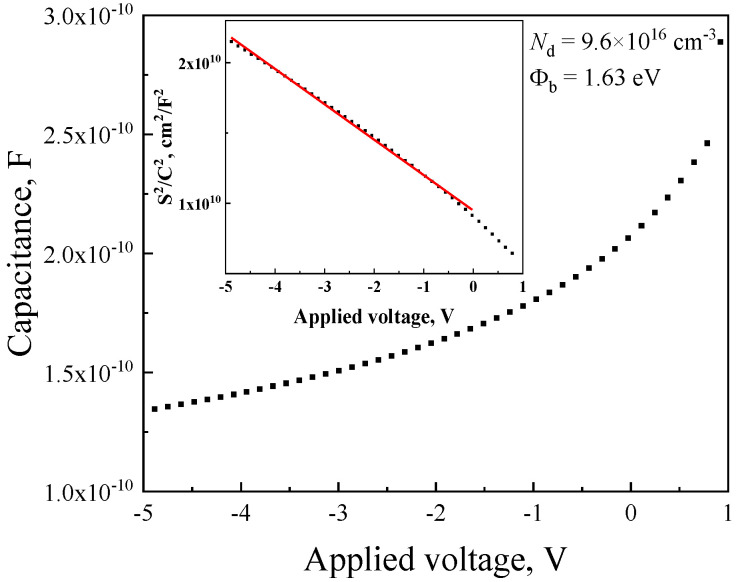
*C*–*V* characteristics of the SBD based on Ni/β-Ga_2_O_3_. Inset shows the dependence of *S*_eff_^2^/*C*^2^ vs. *V*.

The capacitance–voltage (*C*–*V*) characteristics of the Ni/β-Ga_2_O_3_ Schottky structures were measured over the voltage range of −5 to 1 V (Figure 3). The net donor concentration *N*_d_ and Schottky barrier height Φ_b_ values were determined from the analysis of the *S*_eff_^2^/*C*^2^ vs. *V* curves using the following expressions, respectively [36,37]:

(6) $$N_{d}=\;\frac{2}{e\varepsilon_{r}\varepsilon_{0}b},$$

(7) $$\Phi_{b}=\frac{e{N_{d}\varepsilon }_{r}\varepsilon_{0}A}{2},$$

where ε_r_ = 10 arb. un. is the dielectric constant for β-Ga_2_O_3_, ε_0_ is the dielectric constant, *b* is the slope of the linear section of the *C*–*V* characteristic in coordinates of *S*_eff_^2^/*C*^2^ vs. *V*, and *A* is the intersection of the *S*_eff_^2^/*C*^2^ curve with the *y*-axis. The net donor concentration was determined to be 9.6 × 10^16^ cm^−3^, and the Schottky barrier height was found to be 1.63 eV.

**Figure 4:**
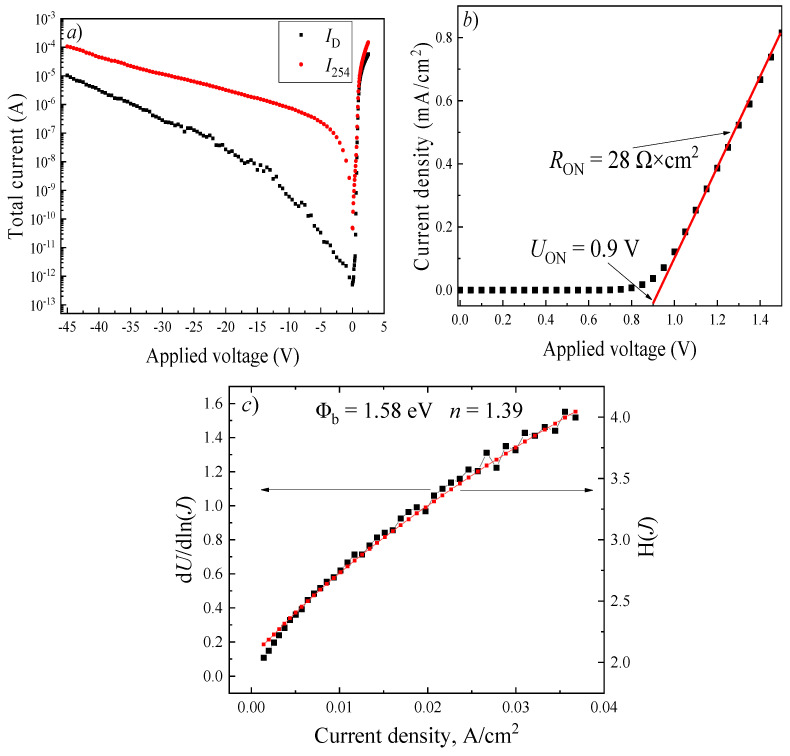
*I*–*V* characteristics of the SBD under dark conditions and under exposure to irradiation at λ = 254 nm and *P* = 620 μW/cm^2^ (**a**); Forward branch of *J*–*V* characteristic of the SBD based on Ni/β-Ga_2_O_3_ (**b**); Cheng’s method plots of d*V*/dln(*J*) vs. *J* and *H*(*J*) vs. J for the SBD based on Ni/β-Ga_2_O_3_ (**c**).

Figure 4a (black curve) shows the current–voltage (*I*–*V*) characteristics of the Schottky barrier diode measured under dark conditions and under illumination at a wavelength of 254 nm. The forward dark current increases from 500 fA to 2.4 μA as the bias voltage rises from 0.01 V to 1 V. According to the thermionic emission (TE) model (Equation (7)), as well as using the Cheung’s method (Equation (8)), the following diode parameters were extracted: saturation current density (*J*ₛ), series resistance (*R*ₛ), ideality factor (*n*), Schottky barrier height (Φ_b_), ON-voltage (*U*_on_) and ON-resistance (*R*_ON_) [33,34]. The obtained values are 6.3 × 10^−16^ A × cm^−2^, 39 kΩ, 1.39, 1.58 eV, 0.9 V, and 28 Ω × cm^2^, respectively. The diode exhibits a high rectification ratio of approximately 9.7 × 10^6^ arb. un. at an applied voltage of ±2 V, confirming the excellent rectifying behavior.

(8) $$J=J_{s}(e^{\frac{eU}{nKT}}-1),$$

(9) $$V=JR_{s}S_{eff}+n\Phi_{b}+\frac{nKT}{e}\times ln(\frac{J}{AT^{2}}),$$

where *R*_s_ is the series resistance of SBD and *H* is the defined function, *S*_eff_ is the effective anode area, A* = 33.5 A/(cm^−2^ × K^−2^) is the Richardson constant, and Φ_b_ is the height of the Schottky barrier.

An increase in the total current of the photodiodes is observed under both reverse and forward bias upon UV illumination at a wavelength of 254 nm with a power density of 620 μW/cm^2^ (Figure 4a, red curve). As the reverse bias is increased from 0 to −45 V, the total current rises from 495 pA to 106 μA, while under forward bias, it reaches 148 μA at +2.5 V. The detectors are capable of operating without an external bias, exhibiting a self-powered mode with an open-circuit voltage (*U*_oc_) of 0.05 V and a short-circuit current (*I*_sc_) of 47 pA.

**Figure 5:**
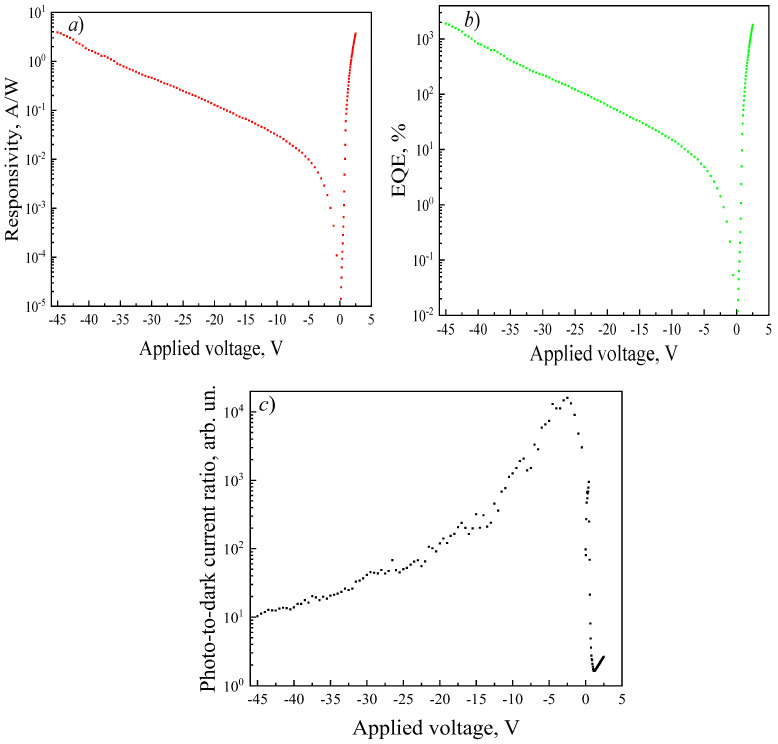
Dependencies of the responsivity (**a**), EQE (**b**), and PDCR (**c**) of the SBD structures on *U* at λ = 254 nm and *P* = 620 µW/cm^2^.

The photoelectric characteristics of the photodiodes were calculated from the measured *I*–*V* data. The responsivity increases from 2 μA/W to 3.87 A/W as the reverse bias is raised from 0 to −45 V and reaches 3.7 A/W at a forward bias of +2.5 V (Figure 5a). This indicates the presence of internal photoconductive gain, as the theoretical responsivity does not exceed 0.21 A/W [38]. The responsivity ratio at the same magnitude of applied voltage ± 2.5 V is *R*(+2.5 V)/*R*(−2.5 V) = 1275 arb. un. A similar trend is observed for the external quantum efficiency (EQE), which increases from 12 × 10^−3^% to 1.8 × 10^3^% under forward bias and to 1.9 × 10^3^% under reverse bias (Figure 5b). The detectivity of the SBD increases from 9.8 × 10^8^ to 4.3 × 10^11^ Hz^0.5^cmW^−1^ as the reverse bias rises to −45 V. The detectivity at a forward bias of 2.5 V was 1.7 × 10^11^ Hz^0.5^cmW^−1^. The maximum PDCR value of 1.6 × 10^4^ arb. un. is observed at a bias of −2.5 V, while further increasing the reverse voltage to −45 V leads to a decrease in the *I*_L_/*I*_D_ ratio to approximately 10 arb. un. due to the sharp rise in dark current (Figure 5c).

**Figure 6:**
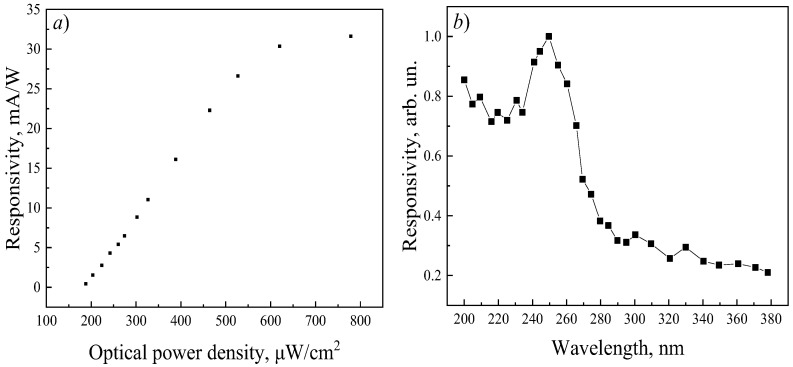
Dependence of the responsivity on the optical power density (**a**) and spectral dependence of the responsivity (**b**) of the Ni/β-Ga_2_O_3_ Schottky barrier diode at *U* = −10 V.

The dependence of responsivity on optical power density was measured for SBD based on Ni/Ga_2_O_3_ (Figure 6a). In the range from 190 to 530 μW/cm^2^, a linear section is observed; further increase in power leads to a smaller growth of responsivity.

Figure 6b shows the spectral dependence of the responsivity of the β-Ga_2_O_3_-based photodiode in the wavelength range of 200–380 nm. A pronounced increase in responsivity is observed starting from approximately 280 nm, reaching a maximum at 255 nm. The residual sensitivity at wavelengths above 260 nm is attributed to defect-related states in the β-Ga_2_O_3_ substrate, which is further supported by the presence of an Urbach tail in the transmission spectrum (Figure 2) [39].

**Figure 7:**
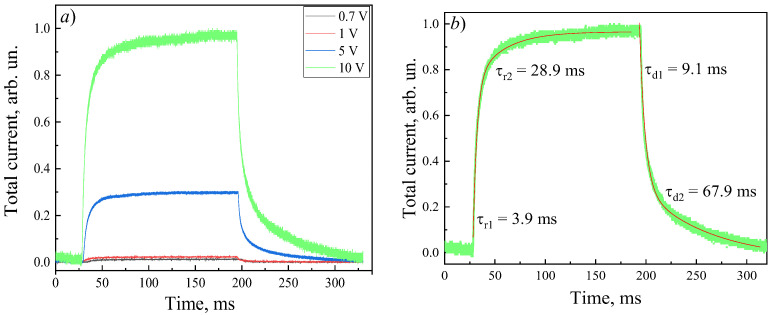
Time-dependent normalized total current of the Ni/β-Ga_2_O_3_ Schottky barrier diode under 255 nm irradiation at different reverse bias voltages (**a**) and at a fixed bias voltage of 10 V (**b**), with biexponential fitting (red curve).

Figure 7 shows the time-dependent photocurrent response of the β-Ga_2_O_3_-based photodiode under periodic ultraviolet illumination at bias voltages of 0.7, 1, 5, and 10 V. As the applied voltage increases, the photocurrent amplitude rises, indicating enhanced sensitivity; however, the response speed decreases. The rise time increases slightly from 19 to 26 ms, while the decay time nearly doubles from 29.8 to 59.5 ms as the bias voltage increases from 0.7 to 10 V (Table 1). Additionally, the transient photocurrent curves display asymmetric rise and decay behaviors, which are associated with carrier trapping and release processes occurring at defect states within the β-Ga_2_O_3_ crystal [40].

The time-dependent photocurrent response of the structures can be approximated by biexponential functions using the following expressions:

(10) $$I\left(t\right)=I_{L}-A_{1}\times exp\left(-\frac{t}{\tau_{r1}}\right)-A_{2}\times exp\left(-\frac{t}{\tau_{r2}}\right),$$

(11) $$I\left(t\right)=I_{D}+B_{1}\times exp\left(-\frac{t}{\tau_{d1}}\right)+B_{2}\times exp\left(-\frac{t}{\tau_{d2}}\right),$$

where *t* is the time; *A*_1_, *A*_2_, *B*_1_, and *B*_2_ are constants; and *τ_r_*_1_, *τ_r_*_2_ and *τ_d_*_1_, *τ_d_*_2_ are the rise and decay time constants of the photoconductivity, respectively. The biexponential behavior indicates the presence of fast (*τ_r_*_1_, *τ_d_*_1_) and slow (*τ_r_*_2_, *τ_d_*_2_) components. The fast-response components of τ_r1_ and τ_d1_ are typically a result of the rapid changes in carrier concentration caused by the generation and recombination of carrier charges during light on/off cycles. The slow-response component of τ_r2_ and τ_d2_ are due to the carrier trapping/releasing from defects [41].

## 4. Discussion

The Schottky barrier height determined from the *I*–*V* characteristics was 1.58 eV, while the value obtained from the *C*–*V* measurements was slightly higher, 1.63 eV. This difference can be attributed to the presence of interface states at the Ni/β-Ga_2_O_3_ interface, which lead to a lower effective barrier height extracted from *I*–*V* analysis compared to the equilibrium value derived from *C*–*V* measurements [42,43].

The sensitivity of the β-Ga_2_O_3_-based detectors to ultraviolet radiation arises from two main mechanisms: band-to-band carrier generation and carrier generation via defect-related states. Band-to-band generation dominates in the deep-UV range, where photon energies exceed the wide bandgap of β-Ga_2_O_3_ (~4.58 eV), while defect states contribute to residual sensitivity at longer wavelengths, as evidenced by the Urbach tail in the transmission spectrum (Figure 2) and the spectral dependence of the responsivity (Figure 7) [44].

In gallium oxide-based photodetectors, three main photogain mechanisms are generally considered: photoconductive gain, barrier-lowering at the Schottky interface, and avalanche multiplication [28,45]. In the present Ni/Ga_2_O_3_ diodes, avalanche multiplication can be excluded, since even at a reverse bias of −45 V the estimated electric field does not exceed 500 kV/cm (assuming the full voltage drops across the depletion region), and the observed photoresponse dynamics are inconsistent with avalanche processes [46]. Therefore, the photoresponse must originate from either barrier-lowering or photoconductive gain. A pronounced photocurrent enhancement is observed under forward bias, where the total photocurrent significantly exceeds the dark current, which is atypical for conventional photodiodes. Notably, the external quantum efficiency reaches 1800% at a forward bias of 2.5 V, where the influence of the Schottky barrier is minimal, yet a strong gain persists. This behavior indicates that barrier-lowering alone cannot account for the observed photoresponse, and that the dominant gain mechanism is photoconductive in nature. The photoconductive gain in these Schottky diode-based detectors is analogous to that in classical photoconductors. It is facilitated by the relatively high series resistance (*R*_s_ = 39 kΩ), which causes a significant portion of the applied voltage to drop across the bulk of the Ga_2_O_3_ layer rather than being confined to the space-charge region near the Schottky contact. Under reverse bias, the contribution of photoconductive gain decreases. This is further confirmed by the responsivity ratio at ±2.5 V, which reaches 1275 arb. un.

However, it should be noted that, according to the literature, the diffusion length of charge carriers in β-Ga_2_O_3_ does not exceed 500 nm [47,48]. In this case, if the ring area formed around the Ni contact is considered as the effective active region, the calculated values of *R* and EQE reach 3.97 × 10^4^ A/W, and 1.9 × 10^7^%, respectively.

The detectors exhibit high responsivity under forward bias, reaching 3.7 A/W at +2.5 V; however, a higher signal-to-noise ratio is observed under reverse bias conditions. This behavior can be explained by the different carrier transport and recombination mechanisms dominating in each regime. Under forward bias, strong carrier injection and the presence of defect-assisted trapping leads to internal gain and enhanced responsivity, but also increase the dark current, thereby reducing the signal-to-noise ratio. In contrast, under reverse bias, the electric field efficiently separates photogenerated carriers and suppresses excess current flow, resulting in lower noise and improved detectivity.

The enhancement in sensitivity comes with a trade-off in response speed (Table 1). The reduction in response speed with increasing applied bias is attributed to the decreased probability of carrier recombination and capture. As carriers traverse the bulk more rapidly due to their higher drift velocity, the likelihood of their being captured by defect states decreases, which in turn slows the recombination of excess carriers [49]. On the other hand, an increase in reverse bias expands the depletion region, activating additional trap states that were not involved at lower bias [50]. The capture and release processes associated with these traps also contribute to the slower transient response. These combined mechanisms explain the observed increase in rise and decay times from 19–26 ms and 29.8–59.5 ms, respectively, as the applied bias increases from 0.7 to 10 V (Table 1, Figure 7). The asymmetric shape of the transient photocurrent curves further indicates the complex dynamics of carrier trapping and emission from defect states in the β-Ga_2_O_3_ crystal [51].

Overall, the results demonstrate that β-Ga_2_O_3_ is a highly functional material, combining pronounced rectifying behavior with strong sensitivity to ultraviolet radiation. The diode characteristics of Ni/β-Ga_2_O_3_ structures ensure low leakage currents and stable operation under dark conditions, while the material exhibits high UV responsivity. This combination of properties, including high photocurrent, significant internal gain, low noise, and the possibility of self-powered operation, makes these devices particularly promising for phototransistor and light-controlled switching applications. The high signal-to-noise ratio under reverse bias and strong responsivity under forward bias enable effective switching between “on” and “off” states, confirming the potential of β-Ga_2_O_3_ for fast-response, energy-efficient optoelectronic devices.

## 5. Conclusions

In this work, we have demonstrated a high-performance deep-ultraviolet photodetector based on a vertical Ni/β-Ga_2_O_3_ Schottky barrier diode fabricated on bulk single-crystal β-Ga_2_O_3_ substrates. The device exhibits excellent rectifying behavior with a Schottky barrier height of 1.63 eV, a low leakage current density of 6.3 × 10^−16^ A × cm^−2^, and a high rectification ratio of ~9.7 × 10^6^ arb. un. The maximum responsivity and external quantum efficiency reach 3.87 A/W and 1.9 × 10^3^%, respectively, indicating internal gain due to carrier trapping and release processes. The detectivity achieves 4.3 × 10^11^ Hz^0.5^cmW^−1^, confirming excellent sensitivity to deep-UV radiation. The device exhibits fast photoconductive response, with rise and decay times of 19 ms and 29 ms, respectively, confirming its high-speed switching performance. These results highlight the potential of bulk β-Ga_2_O_3_ as a functional material for self-powered UV photodetectors combining high responsivity, fast response, and good stability.

## Figures and Tables

**Table 1:** Dependence of the photoelectric characteristics of the Ni/β-Ga_2_O_3_-based photodiode on the applied bias.

Applied Reverse Bias, V	Responsivity, mA/W	EQE, %	*t*_r_, ms	*t*_d_, ms	τ_r1_/τ_r2_, ms	τ_d1_/τ_d2_, ms
0.7	25	17	19	29.8	4.0/25.5	7.1/34.3
1	44	24	29	35	4.4/29.7	6.8/39.7
5	99	48.4	18.3	47.3	4.1/26.2	7.8/51.3
10	305	149	26	59.5	3.9/28.9	9.1/67.9

## Data Availability

All data that support the findings of this study are included within this article.
